# Etiological spectrum and treatment outcome of Obstructive jaundice at a University teaching Hospital in northwestern Tanzania: A diagnostic and therapeutic challenges

**DOI:** 10.1186/1756-0500-4-147

**Published:** 2011-05-23

**Authors:** Phillipo L Chalya, Emmanuel S Kanumba, Mabula Mchembe

**Affiliations:** 1 Department of Surgery, Weill-Bugando University College of Health Sciences, Mwanza, Tanzania; 2 Department of Surgery, Muhimbili University of Health and Allied Sciences, Dar Es Salaam, Tanzania

**Keywords:** Obstructive jaundice, etiological spectrum, treatment outcome, prognostic factors, Tanzania

## Abstract

**Background:**

Obstructive jaundice poses diagnostic and therapeutic challenges to general surgeons practicing in resource-limited countries. This study was undertaken to highlight the etiological spectrum, treatment outcome of obstructive jaundice in our setting and to identify prognostic factors for morbidity and mortality.

**Methods:**

This was a descriptive prospective study which was conducted at Bugando Medical Centre between July 2006 and June 2010. All patients with a clinical diagnosis of obstructive jaundice were, after informed consent for the study, consecutively enrolled into the study. Data were collected using a pre-tested structured questionnaire and analyzed using SPSS computer software version 11.5.

**Results:**

A total of 116 patients were studied. Females outnumbered males by a ratio of 1.3:1. Patients with malignant obstructive jaundice were older than those of benign type. Ca head of pancreas was the commonest malignant cause of jaundice where as choledocholithiasis was the commonest benign cause. Abdominal ultrasound was the only diagnostic imaging done in all patients and revealed dilated intra and extra-hepatic ducts, common bile stones and abdominal masses in 56.2%, 78.9%, 58.1% and 72.4% of the cases respectively. A total of 110 (94.8%) patients underwent surgical treatment and the remaining 6 (5.2%) patients were unfit for surgery. The complication rate was 22.4% mainly surgical site infections. The mean hospital stay and mortality rate were 14.54 days and 15.5% respectively. A low haematocrit and presence of postoperative sepsis were the main predictors of the hospital stay (P < 0.001), whereas age > 60 years, prolonged duration of jaundice, malignant causes and presence of postoperative complications mainly sepsis significantly predicted mortality (P < 0.001).

**Conclusion:**

Obstructive jaundice in our setting is more prevalent in females and the cause is mostly malignant. The result of this study suggests that early diagnosis and treatment plays an important role in the prognosis of patients with obstructive jaundice.

## Background

Obstructive Jaundice is a common surgical problem that occurs when there is an obstruction to the passage of conjugated bilirubin from liver cells to intestine [1]. It is among the most challenging conditions managed by general surgeons and contributes significantly to high morbidity and mortality [2]. As patients with obstructive jaundice have high morbidity and mortality, early diagnosis of the cause of obstruction is very important especially in malignant cases, as resection is only possible at that stage [1-4].

Jaundice due to biliary obstruction may be caused by a heterogeneous group of diseases that include both benign and malignant conditions [5]. The common etiologies of obstructive jaundice have been reported to vary from one centre to another and from one individual to another [5,6]. Obstructive jaundice is not a definitive diagnosis and early investigation to elucidate the precise etiology is of great importance because pathological changes (e.g. secondary biliary cirrhosis) can occur if obstruction is unrelieved [7]. A vast array of invasive and non invasive diagnostic tests is available to diagnose and establish the etiology of surgical obstructive jaundice [4,7]. Invasive tests may cause cholangitis and imaging techniques like computed tomography (CT) scan, PTC, ERCP and MRCP are expensive and are not readily available in most centers in developing countries [7-10], and ultrasonography remains the only diagnostic test available [4,11].

The management of obstructive jaundice poses diagnostic and therapeutic challenges to general surgeons practicing in resource-limited countries [2,12]. Late presentation of the disease coupled with lack of modern diagnostic and therapeutic facilities are among the hallmarks of the disease in developing countries [12]. Surgery in jaundiced patients is associated with a higher risk of postoperative complications compared with surgery in non jaundiced patients [13,14]. These complications primarily consist of septic complications (cholangitis, abscesses, and leakage), hemorrhage, impaired wound healing and renal disorders [14].

The outcome of treatment of obstructive jaundice may be poor especially in developing countries where advanced diagnostic imaging and therapeutic facilities are not readily available in most centers [6]. The mortality and morbidity of biliary obstruction are dependent on the cause of the obstruction, and the assessment of any factors which influence the morbidity and mortality in patients with obstructive jaundice in each society is necessary [6,15]. It has been reported that obstructive jaundice continues to be associated with significant morbidity and mortality despite recent advances both in preoperative diagnosis and postoperative care [15-17]. Understanding factors responsible for increased morbidity and mortality in these patients will better guide appropriate management and lead to improved survival [17].

There is paucity of information regarding the management of obstructive jaundice in our environment as there is no local study which has been done in our setting particularly the study area.

This study was undertaken to describe our own experiences in the management of obstructive jaundice, outlining the etiological spectrum, treatment outcome and prognostic factors for morbidity and mortality in our local setting.

## Methods

This was a prospective study of patients with a clinical diagnosis of obstructive jaundice admitted to the surgical wards of Bugando Medical Centre (BMC) over a 5-year period between July 2006 and June 2010. Bugando Medical Centre is a consultant and teaching hospital for the Weill Bugando University College of Health Sciences and has a bed capacity of 1000. It is situated in Mwanza City, in north-western Tanzania. It is a referral centre for tertiary specialist care for six regions, including Mwanza, Mara, Kagera, Shinyanga and Kigoma. It serves catchments population of approximately 13 million people.

During this study, all patients who were admitted to the surgical wards of BMC with a clinical diagnosis of obstructive jaundice were, after informed written consent, consecutively recruited into the study. Patients with medical jaundice were excluded from the study. Ethical approval to conduct the study was obtained from the WBUCHS/BMC joint institutional ethic review committee before the commencement of the study.

All the patients were subjected to detailed history, clinical examination, laboratory investigations which included the Liver Function tests to see the bilirubin level and the level of serum alkaline phosphatase and ALT and AST. Other laboratory investigations included WBC count, Packed RBC volume, Prothrombin Time, serum creatinine and albumin. Abdominal Ultrasound was the only diagnostic imaging done in all patients to look for the abnormality of intra and extra-hepatic biliary channels, the common bile duct and presence of causative factors like gall tones, tumors, lymph nodes, worms or any abdominal mass. Advanced diagnostic imaging such as CT scan abdomen, ERCP, PTC and MRCP were not done in any of our patients as these facilities are not available at our centre.

The patients were assessed preoperatively, intraoperatively and postoperatively, and the findings were recorded in a pre-tested structured questionnaire. Details that were recorded included patients' biodata, duration of jaundice, cause of obstructive jaundice, laboratory findings, ultra-sonographic findings, treatment modalities, intraoperative findings, post-operative complications, length of hospital stay and mortality. Pre-operative preparations included maintaining good hydration and administration of antibiotics, intravenous dextrose (10%) solution and Vitamin K injections. In anemic patients blood transfusion was also carried out. During surgery intravenous Mannitol was used in all the patients. The nature of surgical procedure carried out depended upon the cause and the findings at the time of surgery. Patients were followed up till discharge or death.

Data analysis was done using SPSS computer software version 11.5. Results were reported as percentages for categorical variables. The variables were compared using the Chi-square test or Fisher's exact test if required for categorical variables. Predictors exhibiting a statistically significant relationship with outcome variables in the univariate analysis (*p*-value less than 0.05) were taken for a multivariate logistic regression analysis to investigate their independence. Odds ratios (OR) and 95% confidence intervals (CI) for OR were calculated. A *p*- value of less than 0.05 was considered statistically significant.

## Results

During the period under study, a total of 116 patients of obstructive jaundice were enrolled. Of these, 50 (43.1%) were males and females were 66 (56.9%) with a male to female ratio of 1:1.3. Their ages ranged from 12 to 78 years with a mean of 56.34 ± 16.42 years. The mean age of patients with benign causes was 42.56 ± 22.12 years (range 12-48 years), while that of malignant causes was 58.64 ± 24.14 years (range 44-78 years). The difference in age distribution of the benign and malignant disease was statistically significant (*P *< 0.001). Both the benign and malignant obstructive jaundice were found to be more commonly amongst the females than males. The male to female ratio for benign obstructive jaundice was 1:2.5, while it was 1:1.4 for the malignant obstructive Jaundice. This difference was statistically significant (*P *< 0.001).

The etiology of obstructive jaundice was benign in 48(41.4%) cases, whereas 68(58.6%) patients had malignant cause. Choledocholithiasis was the commonest cause among the benign group in 30 (62.5%) patients, whereas the commonest tumor among the malignant group was carcinoma of the head of pancreas in 44 (64.7%) patients. Table 1 indicates the different causes of obstructive jaundice.

**Table 1:** Causes of obstructive jaundice

Causes of obstructive jaundice	Number of patients	Percentage
**Benign causes**	**48**	**41.4**
Choledocholithiasis	30	62.5
Biliary strictures	12	25.0
Chronic pancreatitis	3	6.3
Amoebic hepatic abscess	1	2.1
Cause not identified	4	8.3
**Malignant causes**	**68**	**58.6**
Ca head pancreas	44	64.7
Cholangiocarcinoma	8	11.8
Ca gallbladder	6	8.8
Peri-ampulary Ca	6	8.8
Metastatic malignant lymph nodes in the portal hepatic	4	5.9

The duration of illness ranged from 5 to 32 days with a mean and median of 16.34 ± 12.30 days and 16.00 ± 14.22 days respectively. The clinical presentation of obstructive jaundice for both benign and malignant etiology is shown in Table 2.

**Table 2:** Clinical presentation of obstructive jaundice

Clinical feature	Benign	Malignant	Total
Jaundice	48(41.4%)	68(58.6%)	116(100%)
Clay colored stool	42(36.2%)	62(53.4%)	104(89.6%)
Pruritis	40(34.5%)	50(43.1%)	90(77.6%)
Weight loss	5(4.3%)	66(56.9%)	71(61.2%)
Right upper abdominal pain	48(41.4%)	20(17.2%)	68(58.6%)
Scratch marks	30(25.9%)	32 (27.6%)	62(53.4%)
Abdominal mass	1(0.9%)	59(50.9%)	60(51.8%)

Liver function tests were done in all cases and they show raised levels of Bilirubin and Alkaline Phosphatase in both benign and malignant groups. The differences in serum Bilirubin and Alkaline Phosphatase levels in these two groups were not statistically significant (P > 0.05). Serum ALT levels were elevated in 72 (62.1%) of cases. Mean serum bilirubin in patients was 17.1 mg/dL on admission and fell to 2.6 mg/dL after two weeks post surgery (p < 0.05),

Abdominal ultrasound was the only diagnostic imaging done in all patients and revealed dilated intra and extra-hepatic ducts in 56.2% and 78.9% of the cases respectively. Common bile duct stones were detected in only 58.1% of patients. Abdominal masses were detected in 72.4% of the cases. CT scan, ERCP, PTC and MRCP were not performed as they are not available in our institution.

A total of 110 (94.8%) patients underwent open surgical treatment and the remaining 6 (5.2%) patients were found to be unfit for surgery and were treated conservatively. Of the patients who were unfit for surgery, two patients had benign jaundice due to chronic pancreatitis and choledocholithiasis in a sickler patient with severe anemia. The remaining four patients had malignant jaundice. None of our patients had non-surgical procedures such as stricture dilatation and stent placement. Laparoscopic surgery was not done as this facility is not available at our centre. The type of surgical procedures done is shown in Table 3.

**Table 3:** Type of surgical procedure performed (N = 110)

Type of surgical procedure	Number of patients	Percentage
Cholecystojejunostomy	64	58.2
Choledocholithotomy ±Cholecystectomy	18	16.4
Choledochoduodenostomy	10	9.1
Exploratory laparotomy/inoperable	7	6.4
Tumor resection	6	5.5
Hepaticojejunostomy	4	3.4
Drainage of abscess	1	0.9

Major surgeries like pancreatoduodenectomy, hepatectomy and Whipple's procedure were not performed as majority of patients requiring these procedures presented late with advanced disease and the only treatment option was palliative surgery.

Thirty two complications were recorded in 26 patients giving a complication rate of 22.4%. Of these, postoperative wound sepsis was the most common complications. The detail of complications is shown in table 4

**Table 4:** Complications of treatment

Complications	Number of patients	Percentage
Surgical site infections	20	62.5
Coagulopathy	3	9.4
Renal failure	2	6.3
Wound dehiscence	2	6.3
Abdominal abscess/Peritonitis	2	6.3
Hepatic coma	1	3.1
Pneumonia	1	3.1
Leakage of bile	1	3.1

The overall length of hospital stay ranged from 5 days to 44 days (mean 14.54 ± 26 days). 18 patients died giving a mortality rate of 15.5%. The causes of death were postoperative sepsis in 6 (33.3%) patients, advanced malignant in 5 (27.8%) patients, renal failure in 2 (11.1%) patients, disseminated intravascular coagulopathy in 2 (11.1%) patients, associated HIV infection in 2 (11.1%) patients, hepatic coma and cardiac arrest in 1 (5.6%) patient each respectively. A low haematocrit and presence of postoperative sepsis were the main predictors of the hospital stay (P < 0.001), whereas age > 60 years, prolonged duration of jaundice, malignant causes and presence of postoperative complications mainly sepsis significantly predicted mortality (P < 0.001).

## Discussion

Obstructive jaundice poses diagnostic and therapeutic challenges to general surgeons and contributes significantly to high morbidity and mortality [2]. The challenge is even more conspicuous in developing countries like Tanzania where delayed presentation of the disease coupled with lack of modern diagnostic (e.g. CT scan, PTC, ERCP and MRCP) and therapeutic facilities (e.g. T-tubes) are among the hallmarks of the disease [12]. This study was conducted in our local setting to describe our own experiences in the management of this challenging disease; the problem not previously studied at our centre or any other hospital in the country.

The majority of patients in this study had malignant obstructive jaundice which is in agreement with other studies reported elsewhere [1,4,6,8,18,19], but in contrast to Bekele *et al *[12] in Ethiopia who reported benign obstructive jaundice (choledocholithiasis) as the most common cause of obstructive jaundice. In our study, carcinoma of the head of pancreas was the commonest cause of malignant obstructive jaundice while choledocholithiasis was the commonest benign cause. Similar observation was also noted by others [8,18,19]. Sharm & Ahuja [4] reported carcinoma of the gall bladder as the most common cause of malignant obstructive jaundice. Also, studies in Saudi Arabia and Yemen reported *Ascariases Lumbricoides *to be frequently associated with disease of biliary tract resulting in obstruction [20]. These observations reflect differences in etiological spectrum from one centre to another.

In this study, both the benign and malignant obstructive jaundice were found to be more common in females than in males, which is in conformity with the results of other researchers [1,4,8,18]. Female preponderance in both the benign and malignant obstructive jaundice has been ascribed to high prevalence of gall stones in them which is reported to be a risk factor for many benign and malignant conditions causing biliary obstruction[21-23].

Most of the patients with benign obstructive jaundice in our study were in younger age group while malignant causes were in elder age group. The incidence of malignant obstructive jaundice in patients of older age group was also reported by others [6,8,18,19].

All patients in our study presented with jaundice associated with clay colored stool (89.6%), pruritis (77.6%), weight loss (61.2%), right upper abdominal pain (58.6%), scratch marks (53.6%) and palpable abdominal mass (51.8%). Clay colored stool, weight loss and palpable abdominal mass were more common in malignant obstructive jaundice, whereas right upper abdominal pain was more common in benign obstructive jaundice. Pruritis and scratch marks were seen equally in both the benign and malignant cases. Similar clinical pattern was also reported by others [1,8,12,18]. The presence of right upper abdominal pain in patients with malignant obstructive jaundice is probably due to advanced disease. The abdominal mass was appreciated in 50.9% of the patients with malignancy due to the local spread of tissues and no mass was palpable in cases of choledocholithiasis or any other benign condition thus supporting the 'Courvoisier's law [8,19,24].

The biochemical investigations done was liver function tests which showed high serum bilirubin and alkaline phosphatase levels. The difference in serum bilirubin and alkaline phosphatase levels between benign and malignant obstructive jaundice was not statistically significant. This finding is in agreement with other studies [8,18]. In one study by Cheema *et al *[25], the values of bilirubin and alkaline phosphatase were found to be higher in the malignant cases.

Patients with obstructive jaundice have increased levels of serum bilirubin and most of the complications in the preoperative and postoperative period in obstructive jaundice have been attributed to hyperbilirubinemia [26]. Following definitive surgery to relieve obstruction, there is a significant fall in serum bilirubin levels. The time taken for achieving a significant drop in serum bilirubin levels may vary from 4 days to 3 weeks after definitive surgery [27]. Afify et al [28] reported a significant fall in serum bilirubin levels. This agrees to the results of our study. Definitive surgery to relieve obstructive jaundice plays a greater role in reversing the pathophysiological disturbances associated with obstructive jaundice and thus reduce postoperative morbidity and mortality [26-28].

In places where advanced diagnostic imaging (e.g. CT scan, ERCP, PTC and MRCP) are not available as in most centers in developing countries, abdominal ultrasound has been reported to be the first and best initial imaging procedure in patients who have obstructive jaundice and shows reasonable sensitivity and specificity to identify causes of obstruction in obstructive jaundice [4,11,12,19,25]. Abdominal ultrasound was the only diagnostic imaging done in all our patients as most of the advanced radiological tests such as abdominal CT scan, ERCP, PTC and MRCP are not available at our centre. This observation is common in most studies in developing countries [12,11,29].

In our study, the majority of patients with malignant obstructive jaundice underwent palliative surgery mainly by bypass surgery, whereas the majority of patients with benign obstructive jaundice underwent curative surgery. Similar treatment pattern was also reported by Mohammed *et al *[1]. High incidence of palliative surgery in patients with malignant obstructive jaundice is due to delayed presentation for treatment as a result the majority of patients with malignant conditions report to hospital very late when the disease is in advanced stage, and the only option is palliative surgery.

Since surgery in patients with jaundice is thought to increase the risk of postoperative complications, preoperative biliary drainage {PBD) was introduced as means of reversing the pathophysiological disturbance seen in jaundiced patients and has been advocated before curative tumor resection by some surgeons to decrease the complications of biliary surgery in the jaundiced patients [30]. Early studies showed a reduction in morbidity [16]. However, more recently the focus has shifted towards the negative effects of drainage, such as an increase increased incidence of postoperative morbidity [mostly infectious complications] and postoperative mortality [31]. Whether biliary drainage should always be performed in jaundiced patients remains controversial. The use of PBD has not been evaluated in our setting and therefore none of our patient in this series underwent PBD. It is therefore recommended that, a prospective randomized trial addressing the effects of PBD, and thus solving the longstanding controversy whether or not PBD should be routinely performed in jaundiced patients prior to a surgery, is highly indicated in our setting so as to justify its benefits.

Traditionally, T-tube placement after common biliary duct exploration has long been a standard surgical practice for choledocholithiasis, but potential of complications exists with this therapeutic modality [29,31]. These include sepsis, dislodgement of tube, obstruction and/or fracture of tube, leakage of bile, incomplete evacuation, recurrent stone and patient may have to carry it for several weeks before removal. All of these lead to prolong length of hospital stay [31,32]. The use of T-tube has been challenged by recent clinical trials which advocated primary closure of common bile duct after open Choledocholithotomy for Choledocholithiasis [31-34]. In our study, primary closure of common bile duct after open Choledocholithotomy for Choledocholithiasis was used in the majority of patients as T-tubes are not available in our centre.

In our study, complication rate was recorded in 22.4% of cases. Of this, surgical site infection was the most common postoperative complications and the most found organisms were *E. coli, Klebsiella and Proteus*. Similar bacterial profile was also found in other studies [35]. Postoperative wound infections in biliary surgery has been reported in literature to be due to contamination by gram negative enteric aerobes like *E. coli, Klebsiella and Proteus *produced by opening the biliary tract in patients with bactibilia [36-38]. High incidence of these organisms in our study may be due to contamination of the wound during the surgical procedure.

The figure for the overall duration of hospital stay in our study was higher than that reported in other studies [6,13,14]. The predictors of the length of hospital stay were low haematocrit and presence of postoperative sepsis.

Surgical procedure in patients with obstructive jaundice has been reported to be associated with significant mortality compared with surgery in non jaundiced patients [13-15,31]. Mortality rates of between eight and 33% have been reported for surgery to relieve bile duct obstruction [13,16,17].

Several factors including elder age group, duration of jaundice, malignant causes, high levels of bilirubin and presence postoperative complications (e.g. sepsis, coagulopathy, hepatic coma and renal failure) have been reported in literature to be associated with high mortality rate in these patients [6,13,15]. Our mortality rate of 15.5% was higher that reported in an Iranian study by Mehrdad *et al *[6]. The predictors of mortality in our study were age > 60 years, prolonged duration of jaundice, malignant causes and presence postoperative complications mainly sepsis.

The high morbidity and mortality rates in this study are attributed to delayed presentation of disease and lack of modern diagnostic and therapeutic facilities seen in developed world. Findings from this study is a typical example of diagnostic and therapeutic challenges seen in most developing countries where delayed presentation of the disease coupled with lack of modern diagnostic (e.g. CT scan, PTC, ERCP and MRCP) and therapeutic facilities (e.g. T-tubes) are among the hallmarks of the disease.

## Conclusion

Obstructive jaundice is a common surgical problem in our setting and poses diagnostic and therapeutic challenges. It is more common among females with malignant causes being more prevalent. The benign jaundice is seen in young patients while malignant causes in elder age group. Carcinoma of the head of pancreas is the commonest malignant cause of jaundice whereas stones in the bile duct the commonest benign etiology. Most of patients with malignant obstructive jaundice present late with advanced disease and the only treatment modality for these patients was palliative surgery. The majority of patients with Choledocholithiasis were treated with Choledocholithotomy and primary closure of the common bile duct. The result of this study suggests that early diagnosis and treatment plays an important role in the prognosis of patients with obstructive jaundice. Primary closure of the common bile duct is safe and cost effective alternative to routine T-tube drainage after open Choledocholithotomy and associated with low complication rates.

## Abbreviations

ALT: Alanine aminotransferase; AST: Aspartate aminotransferase; CT scan: Computed Tomography scan; ERCP: Endoscopic Retrograde Cholangiopancreatography; MRCP: Magnetic Resonance Cholangiopancreatography; PTC: Percutaneous Transhepatic Cholangiopancreatography

## Competing interests

The authors declare that they have no competing interests.

## Authors' contributions

PLC conceived the study and did the literature search, coordinated the write-up, editing and submission of the article. ESK and MM participated in the writing of the manuscript and editing. All the authors read and approved the final manuscript.
